# HB-PLS: A statistical method for identifying biological process or pathway regulators by integrating Huber loss and Berhu penalty with partial least squares regression

**DOI:** 10.48130/FR-2021-0006

**Published:** 2021-03-30

**Authors:** Wenping Deng, Kui Zhang, Cheng He, Sanzhen Liu, Hairong Wei

**Affiliations:** 1 College of Forest Resources and Environmental Science, Michigan Technological University, Houghton, Michigan 49931, United States of America; 2 Department of Mathematical Science, Michigan Technological University, Houghton, Michigan 49931, United States of America; 3 Department of Plant Pathology, Kansas State University, Manhattan, Kansas 66506, United States of America

**Keywords:** Huber loss, Berhu penalty, partial least squares regression, Huber-Berhu partial least squares, pathway regulators

## Abstract

Gene expression data features high dimensionality, multicollinearity, and non-Gaussian distribution noise, posing hurdles for identification of true regulatory genes controlling a biological process or pathway. In this study, we integrated the Huber loss function and the Berhu penalty (HB) into partial least squares (PLS) framework to deal with the high dimension and multicollinearity property of gene expression data, and developed a new method called HB-PLS regression to model the relationships between regulatory genes and pathway genes. To solve the Huber-Berhu optimization problem, an accelerated proximal gradient descent algorithm with at least 10 times faster than the general convex optimization solver (CVX), was developed. Application of HB-PLS to recognize pathway regulators of lignin biosynthesis and photosynthesis in *Arabidopsis thaliana* led to the identification of many known positive pathway regulators that had previously been experimentally validated. As compared to sparse partial least squares (SPLS) regression, an efficient method for variable selection and dimension reduction in handling multicollinearity, HB-PLS has higher efficacy in identifying more positive known regulators, a much higher but slightly less sensitivity/(1-specificity) in ranking the true positive known regulators to the top of the output regulatory gene lists for the two aforementioned pathways. In addition, each method could identify some unique regulators that cannot be identified by the other methods. Our results showed that the overall performance of HB-PLS slightly exceeds that of SPLS but both methods are instrumental for identifying real pathway regulators from high-throughput gene expression data, suggesting that integration of statistics, machine leaning and convex optimization can result in a method with high efficacy and is worth further exploration.

## INTRODUCTION

In a gene regulatory network (GRN), a node corresponds to a gene and an edge represents a directional regulatory relationship between a transcription factor (TF) and a target gene. Understanding the regulatory relationships among genes in GRNs can help elucidate the various biological processes and underlying mechanisms in a variety of organisms. Although experiments can be conducted to acquire evidence of gene regulatory interactions, these are labor-intensive and time-consuming. In the past two decades, the advent of high-throughput technologies including microarray and RNA-Seq, have generated an enormous wealth of transcriptomic data. As the data in public repositories grows exponentially, computational algorithms and tools utilizing gene expression data offer a more time- and cost-effective way to reconstruct GRNs. To this end, efficient mathematical and statistical methods are needed to infer qualitative and quantitative relationships between genes.

Many methods have been developed to reconstruct GRNs, each employing different theories and principles. The earliest methods include differential equations^[1]^, Boolean networks^[2]^, stochastic networks^[3]^, Bayesian^[4,5]^ or dynamic Bayesian networks (BN)^[6,7]^, and ordinary differential equations (ODE)^[8]^. Some of these methods require time series datasets with short time intervals, such as those generated from easily manipulated single cell organisms (e.g. bacteria, yeast etc.) or mammalian cell lines^[9]^. For this reason, most of these methods are not suitable for gene expression data, especially time series data involving time intervals on the scale of days, from multicellular organisms like plants and mammals (except cell lines).

In general, the methods that are useful for building gene networks with non-time series data generated from higher plants and mammals include ParCorA^[10]^, graphical Gaussian models (GGM)^[11]^, and mutual information-based methods such as Relevance Network (RN)^[12]^, Algorithm for the Reconstruction of Accurate Cellular Networks (ARACNE)^[13]^, C3NET^[14]^, maximum relevance/minimum redundancy Network (MRNET)^[15]^, and random forests^[16,17]^. Most of these methods are based on the information-theoretic framework. For instance, Relevance Network (RN)^[18]^, one of the earliest methods developed, infers a network in which a pair of genes are linked by an edge if the mutual information is larger than a given threshold. The context likelihood relatedness (CLR) algorithm^[19]^, an extension of RN, derives a score from the empirical distribution of the mutual information for each pair of genes and eliminates edges with scores that are not statistically significant. ARACNE is similar to RN; however, ARACNE makes use of the data processing inequality (DPI) to eliminate the least significant edge of a triplet of genes, which decreases the false positive rate of the inferred network. MRNET^[20]^ employs the maximum relevance and minimum redundancy feature selection method to infer GRNs. Finally, triple-gene mutual interaction (TGMI) uses condition mutual information to evaluate triple gene blocks to infer GRNs^[21]^. Information theory-based methods are used extensively for constructing GRNs and for building large networks because they have a low computational complexity and are able to capture nonlinear dependencies. However, there are also disadvantages in using mutual information, including high false-positive rates^[22]^ and the inability to differentiate positive (activating), negative (inhibiting), and indirect regulatory relationships. Reconstruction of the transcriptional regulatory network can be implemented by the neighborhood selection method. Neighborhood selection^[23]^ is a sub-problem of covariance selection. Assume $ \Gamma  $ is a set containing all of the variables (genes), the neighborhood $ {ne}_{a} $ of a variable $  a\in \Gamma  $ is the smallest subset of $  \Gamma \backslash \left\{a\right\} $ such that, given all variables in $ {ne}_{a}\,  $ , variable $ a $ is conditionally independent of all remaining variables. Given $ n $ i.i.d. observations of $ \Gamma  $, neighborhood selection aims to estimate the neighborhood of each variable in $ \Gamma  $ individually. The neighborhood selection problem can be cast as a multiple linear regression problem and solved by regularized methods.

Following the differential equation in^[24]^, the expression levels of a target gene $ y $ and the expression levels of the TF genes $ x $ form a linear relationship:

1 $
{y}_{i}={\beta }_{0}+{x}_{i}^{T}\beta +{\varepsilon }_{i}\;\;\;   i=\mathrm{1,2},\dots ,n 
$

where $ n $ is the number of samples, $  {x}_{i}={({x}_{i1},\dots ,{x}_{ip})}^{T} $ is the expression level of $ p $ TF genes, and $  {y}_{i} $ is the expression level of the target gene in sample $ i $. $ {\beta }_{0} $ is the intercept and ${\boldsymbol{\beta }} ={({\beta }_{1},\cdots ,{\beta }_{p})}^{T}$ are the associated regression coefficients; if any $  {\beta }_{j}\ne 0 $ $  (j=1,\cdots ,p) $, then TF gene $ j $ regulates target gene $ i $. $  {\{\varepsilon }_{i}\} $ are independent and identically distributed random errors with mean 0 and variance $ {\sigma }^{2} $. The method to get an estimate of $\boldsymbol{\beta }$ and $ {\beta }_{0} $ is to transform this statistical problem to a convex optimization problem:

2 $
{\boldsymbol{\beta }}={argmin}_{\boldsymbol{\beta }} f\left({\boldsymbol{\beta }}\right)={argmin}_{\boldsymbol{\beta }}{\sum }_{i=1}^{n}L\left({y}_{i}-{{{\beta}} }_{0}-{x}_{i}^{T}{\boldsymbol{\beta }}\right) +\lambda P\left({\boldsymbol{\beta }}\right) 
$

where $  L(\cdot ) $ is a loss function, $  P(\cdot ) $ is a penalization function, and $  \lambda >0 $ is a tuning parameter which determines the importance of penalization. Different loss functions, penalization functions, and methods for determining $ \lambda  $ have been proposed in the literature. Ordinary least squares (OLS) is the simplest method with a square loss function $L({y}_{i}-{\beta }_{0}-{x}_{i}^{T}{\boldsymbol{\beta }})={({y}_{i}-{\beta }_{0}-{x}_{i}^{T}{\boldsymbol{\beta }})}^{2}$ and no penalization function. The OLS estimator is unbiased^[25]^. However, since it is common for the number of genes, $p$, to be much larger than the number of samples, $ n $, (i.e. $  p\gg n) $ in any given gene expression data set, there is no unique solution for OLS. Even when $n > p$, OLS estimation features high variance. To tackle these problems, ridge regression^[26]^ adds a $\ell _{2}$ penalty, $P\left({\boldsymbol{\beta }}\right)={\sum }_{j=1}^{p}{\beta }_{j}^{2}$, on the coefficients which introduces a bias but reduces the variance of the estimated, $ \hat{\beta } $. In ridge regression, there is a unique solution even for the $  p>n $ case. Least absolute shrinkage and selection operator (LASSO)^[27]^ is similar to ridge regression, except the $\ell _{2}$ penalty in ridge regression is replaced by the $\ell_{1}$ penalty, $P\left({\boldsymbol{\beta }}\right)={\sum }_{j=1}^{p}\left|{\beta }_{j}\right|$.

The main benefit of least absolute shrinkage and selection operator (LASSO) is that it performs variable selection and regularization simultaneously thereby generating a sparse solution, a desirable property for constructing GRNs. When LASSO is used for selecting regulatory TFs for a target gene, there are two potential limitations. First, if several TF genes are correlated and have large effects on the target gene, LASSO has a tendency to choose only one TF gene while zeroing out the other TF genes. Second, some studies^[28]^ state that LASSO does not have oracle properties; that is, it does not have the capability to identify the correct subset of true variables or to have an optimal estimation rate. It is claimed that there are cases where a given $ \lambda  $ that leads to optimal estimation rate ends up with an inconsistent selection of variables. For the first limitation, Zou and Hastie^[29]^ proposed elastic net, in which the penalty is a mixture of LASSO and ridge regressions: $P\left({\boldsymbol{\beta }}\right)=\alpha {\sum }_{j=1}^{p}\left|{\beta }_{j}\right|+\frac{1-\alpha }{2}{\sum }_{j=1}^{p}{\beta }_{j}^{2}\;{\myriadfont\text{,}}$ where $  \alpha  (0<\alpha <1) $ is called the elastic net mixing parameter. When $  \alpha =1\,{\myriadfont\text{,}} $ the elastic net penalty becomes the LASSO penalty; when $  \alpha =0 $, the elastic net penalty becomes the ridge penalty. For the second limitation, adaptive LASSO^[28]^ was proposed as a regularization method, which enjoys the oracle properties. The penalty function for adaptive LASSO is: $P\left({\boldsymbol{\beta }}\right)={\sum }_{j=1}^{p}{\hat{w}}_{j}\left|{\beta }_{j}\right| \;{\myriadfont\text{,}}$ where adaptive weight $  {\hat{w}}_{j}=\frac{1}{{\left|{\hat{\beta }}_{ini}\right|}^{\gamma }}\;{\myriadfont\text{,}} $ and $  \left|{\hat{\beta }}_{ini}\right| $ is an initial estimate of the coefficients obtained through ridge regression or LASSO; $ \gamma  $ is a positive constant, and is usually set to 1. It is evident that adaptive LASSO penalizes more those coefficients with lower initial estimates.

It is well known that the square loss function is sensitive to heavy-tailed errors or outliers. Therefore, adaptive LASSO may fail to produce reliable estimates for datasets with heavy-tailed errors or outliers, which commonly appear in gene expression datasets. One possible remedy is to remove influential observations from the data before fitting a model, but it is difficult to differentiate true outliers from normal data. The other method is to use robust regression. Wang et al.^[30]^ combined the least absolute deviation (LAD) and weighted LASSO penalty to produce the LAD-LASSO method. The objective function is:

3 $
{\sum }_{i=1}^{n}\left|{y}_{i}-{\beta }_{0}-{x}_{i}^{T}{\boldsymbol{\beta }}\right|+\lambda {\sum }_{j=1}^{p}{\hat{w}}_{j}\left|{\beta }_{j}\right|
$

With this LAD loss, LAD-LASSO is more robust than OLS to unusual $ y $ values, but it is sensitive to high leverage outliers. Moreover, LAD estimation degrades the efficiency of the resulting estimation if the error distribution is not heavy tailed^[31]^. To achieve both robustness and efficiency, Lambert-Lacroix and Zwald 2011^[32]^, proposed Huber-LASSO, which combined the Huber loss function and a weighted LASSO penalty. The Huber function (see Materials and Methods) is a hybrid of squared error for relatively small errors and absolute error for relatively large ones. Owen 2007^[33]^ proposed the use of the Huber function as a loss function and the use of a reversed version of Huber’s criterion, called Berhu, as a penalty function. For the Berhu penalty (see Materials and Methods), relatively small coefficients contribute their $\ell _{1}$ norm to the penalty while larger ones cause it to grow quadratically. This Berhu penalty sets some coefficients to 0, like LASSO, while shrinking larger coefficients in the same way as ridge regression. In^[34]^, the authors showed that the combination of the Huber loss function and an adaptive Berhu penalty enjoys oracle properties, and they also demonstrated that this procedure encourages a grouping effect. In previous research, the authors solved a Huber-Berhu optimization problem using CVX software^[33−35]^, a Matlab-based modeling system for convex optimization. CVX turns Matlab into a modeling language, allowing constraints and objectives to be specified using standard Matlab expression syntax. However, since CVX is slow for large datasets, a proximal gradient descent algorithm was developed for the Huber-Berhu regression in this study, which runs much faster than CVX.

Reconstruction of GRNs often involves ill-posed problems due to high dimensionality and multicollinearity. Partial least squares (PLS) regression has been an alternative to ordinary regression for handling multicollinearity in several areas of scientific research. PLS couples a dimension reduction technique and a regression model. Although PLS has been shown to have good predictive performance in dealing with ill-posed problems, it is not particularly tailored for variable selection. Sæbø et al. 2007^[36]^ first proposed the soft-threshold-PLS (ST-PLS), in which the $\ell _{1}$ penalty is used for PLS loading weights of multiple latent components. Such a method is especially applicable for classification and variable selection when the number of variables is greater than the number of samples. Chun and Keleş 2010^[37]^ proposed a similar sparse PLS regression for simultaneous dimension reduction and variable selection. Both the methods from Sæbø et al. 2007 and Chun and Keleş 2010 used the same $\ell _{1}$ penalty for PLS loading weights. Lê Cao et al. 2008^[38]^ also proposed a sparse PLS method for variable selection when integrating omics data. They added sparsity into PLS with a LASSO penalization combined with singular value decomposition (SVD) computation. In this study, the Huber loss function and the Berhu penalty function were embedded into a PLS framework. Real gene data was used to demonstrate that this approach is applicable for the reconstruction of GRNs.

## MATERIALS AND METHODS

### High-throughput gene expression data

The lignin pathway analysis used an *Arabidopsis* wood formation compendium dataset containing 128 Affymetrix microarrays pooled from six experiments (accession identifiers: GSE607, GSE6153, GSE18985, GSE2000, GSE24781, and GSE5633 in NCBI Gene Expression Omnibus (GEO) (http://www.ncbi.nlm.nih.gov/geo/)). These datasets were originally obtained from hypocotyledonous stems under short-day conditions known to induce secondary wood formation^[39]^. The original CEL files were downloaded from GEO and preprocessed using the affy package in Bioconductor (https://www.bioconductor.org/) and then normalized with the robust multi-array analysis (RMA) algorithm in affy package. This compendium data set was also used in our previous studies^[40]^. The maize B73 compendium data set used for predicting photosynthesis light reaction (PLR) pathway regulators was downloaded from three NCBI databases: (1) the sequence read archive (SRA) (https://www.ncbi.nlm.nih.gov/sra), 39 leaf samples from ERP011838; (2) Gene Expression Omnibus (GEO), 24 leaf samples from GSE61333, and (3) BioProject (https://www.ncbi.nlm.nih.gov/bioproject/), 36 seedling samples from PRJNA483231. This compendium is a subset of that used in our earlier co-expression analysis^[41]^. Raw reads were trimmed to remove adaptors and low-quality base pairs via Trimmomatic (v3.3). Clean reads were aligned to the B73Ref3 with STAR, followed by the generation of normalized FPKM (fragments per kb of transcript per million reads) using Cufflinks software (v2.1.1)^[42]^.

### Huber and Berhu functions

In estimating regression coefficients, the square loss function is well suited if $ {y}_{i} $ follows a Gaussian distribution, but it gives a poor performance when $ {y}_{i} $ follows a heavy-tailed distribution or there are outliers. On the other hand, the least absolute deviation (LAD) loss function is more robust to outliers, but the statistical efficiency is low when there are no outliers in the data. The Huber function, introduced in^[43]^, is a combination of linear and quadratic loss functions. For any given positive real $ M $ (called shape parameter), the Huber function is defined as:

4 $
\begin{array}{c}{H}_{M}\left({\textit z}\right)=\left\{\begin{array}{cc}{{\textit z}}^{2}& \left|{\textit z}\right|\le M\\ 2M\left|{\textit z}\right|-{M}^{2}& \left|{\textit z}\right|>M\end{array}\right.\end{array}
$

**Figure 1:**
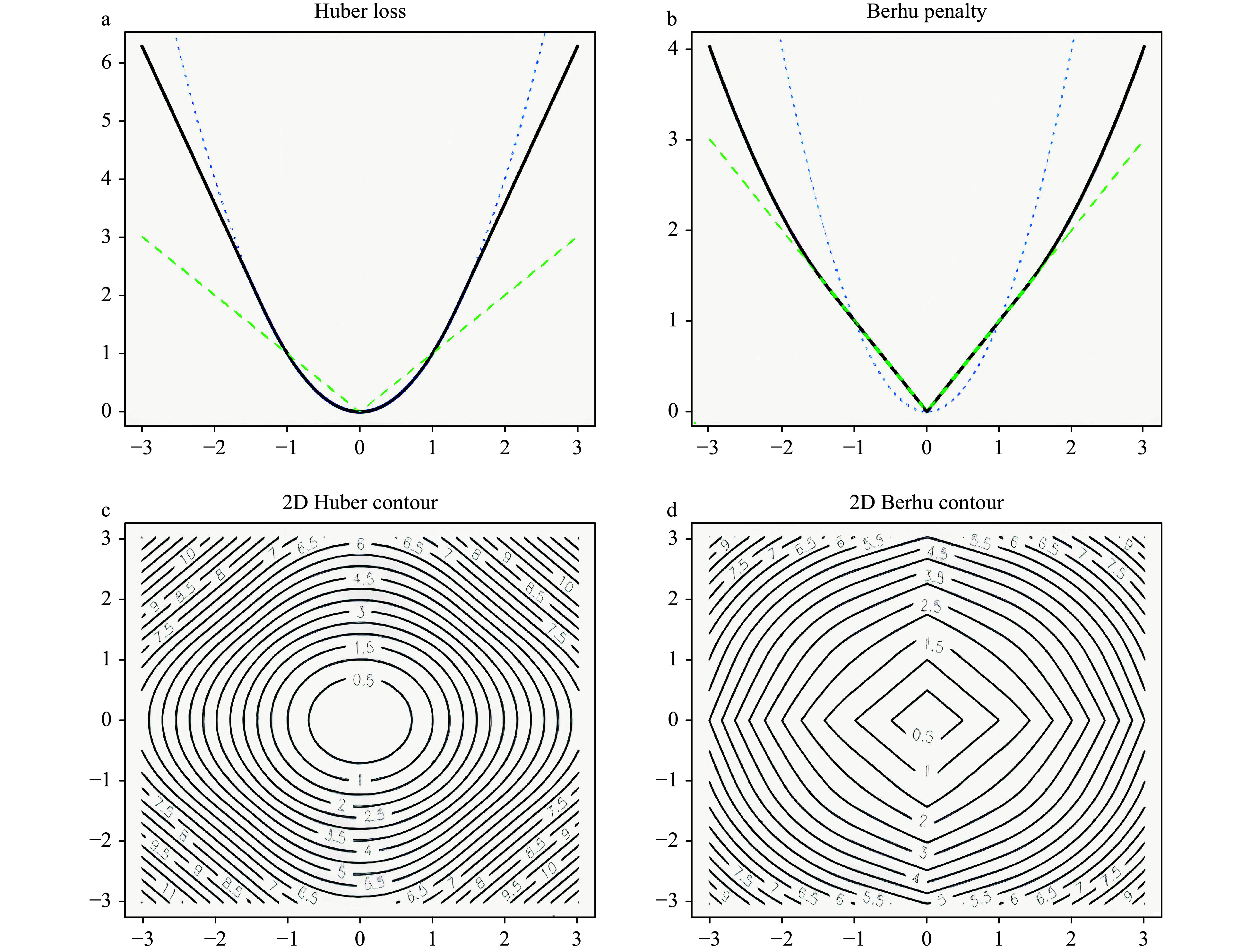
Huber loss function (a) and Berhu penalty function (b); The 2D contours of Huber loss function (c) and Berhu penalty function (d).

This function is quadratic for small $ {\textit z} $ values but grows linearly for large values of $ {\textit z} $. The parameter $ M $ determines where the transition from quadratic to linear takes place (Fig. 1a). In this study, the default value of $ M $ was set to be one tenth of the interquartile range (IRQ), as suggested by^[44]^. The Huber function is a smooth function with a derivative function:

5 $
\begin{array}{c}{H}_{M}'\left({\textit z}\right)=\left\{\begin{array}{cc}2{\textit z}& \left|{\textit z}\right|\le M\\ 2M \; sign\left({\textit z}\right)& \left|{\textit z}\right|>M\end{array}\right.\end{array}
$

The ridge regression uses the quadratic penalty on regression coefficients, and it is equivalent to putting a Gaussian prior on the coefficients. LASSO uses a linear penalty on regression coefficients, and this is equivalent to putting a Laplace prior on the coefficients. The advantage of LASSO over ridge regression is that it implements regularization and variable selection simultaneously. The disadvantage is that, if a group of predictors is highly correlated, LASSO picks only one of them and shrinks the others to zero. In this case, the prediction performance of ridge regression dominates the LASSO. The Berhu penalty function, introduced in Owen 2007^[33]^, is a hybrid of the quadratic penalty and LASSO. It gives a quadratic penalty to large coefficients while giving a linear penalty to small coefficients, as shown in Fig. 1b. The Berhu function is defined as:

6 $
\begin{array}{c}{B}_{M}\left({\textit z}\right)=\left\{\begin{array}{cc}\left|{\textit z}\right|& \left|{\textit z}\right|\le M\\ \dfrac{{{\textit z}}^{2}+{M}^{2}}{2M}& \left|{\textit z}\right|>M\end{array}\right.\end{array} 
$

The shape parameter $ M $ was set to be the same as that in the Huber function. As shown in Fig. 1b, the Berhu function is a convex function, but it is not differentiable at $  {\textit z}=0 $. The 2D contours of Huber and Berhu functions are shown in Fig. 1c and Fig. 1d, respectively. When the Huber loss function and the Berhu penalty were combined, an objective function, as referred as the Huber-Berhu function, was obtained, as shown below.

7 $
\begin{array}{c}f\left({\boldsymbol{\beta }}\right)={\displaystyle\sum }_{i=1}^{n}{H}_{M}({y}_{i}-\beta_0-{x}_{i}^{T}{\boldsymbol{\beta}} )+\lambda {\displaystyle\sum }_{j=1}^{p}{B}_{M}\left({\beta }_{j}\right)\end{array}
$

**Figure 2:**
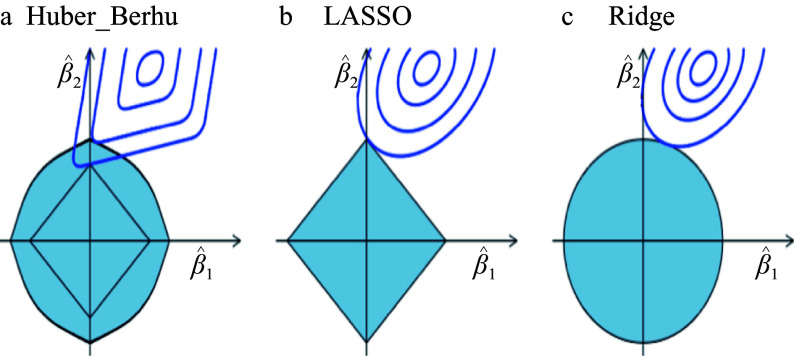
Estimation picture for the Huber-Berhu regression (a) when least absolute shrinkage and selection operator (LASSO) (b) and ridge (c) regressions are used as a comparison.

The estimation of coefficients using the Huber-Berhu objective (Fig. 2a), LASSO (Fig. 2b), and the ridge (Fig. 2c) regressions provided some insights. The Huber loss corresponds to the rotated, rounded rectangle contour in the top right corner, and the center of the contour is the solution of the un-penalized Huber regression. The shaded area is a map of the Berhu constraint where a smaller $ \lambda  $ corresponds to a larger area. The estimated coefficient of the Huber-Berhu regression is the first place the contours touch the shaded area; when $ \lambda  $ is small, the touch point is not on the axes, which means the Huber-Berhu regression behaves more like the ridge regression, which does not generate a sparse solution. When $ \lambda  $ increases, the correspondent shaded area changes to a diamond, and the touch point is more likely to be located on the axes. Therefore, for large $ \lambda  $, the Huber-Berhu regression behaves like LASSO, which can generate a sparse solution.

### The algorithm to solve the Huber-Berhu regression

Since the Berhu function is not differentiable at $ {\textit z}=0 $, it is difficult to use the gradient descent method to solve equation (4). Although we can use the general convex optimization solver CVX^[35]^ for a convex optimization problem, it is too slow for real biological applications. Therefore, a proximal gradient descent algorithm was developed to solve equation (4). Proximal gradient descent is an effective algorithm to solve an optimization problem with decomposable objective function. Suppose the objective function can be decomposed as $  f\left({\textit z}\right)=g\left({\textit z}\right)+h\left({\textit z}\right) $, where $  g\left({\textit z}\right) $ is a convex differentiable function and $  h\left({\textit z}\right) $ is a convex non-differentiable function. The idea behind the proximal gradient descent^[45]^ method is to make a quadratic approximation to $  g\left({\textit z}\right) $ and leave $  h\left({\textit z}\right) $ unchanged. That is:

$f\left( {\textit z} \right) = g\left( {\textit z} \right) + h\left( {\textit z} \right) \approx g\left( {\textit z} \right) + \nabla g{\left( x \right)^T}\left( {{\textit z} - x} \right) + \frac{1}{{2t}}\left\| {{\textit z} - x} \right\|_2^2 + h\left( {\textit z} \right)$

At each step, $ x $ is updated by the minimum of the right side of above formula.

\begin{equation*}\begin{split}
{x^ + } =\;& argmi{n_{\textit z}}\;g\left( x \right) + \nabla g{\left( {\textit z} \right)^T}\left( {{\textit z} - x} \right) + \frac{1}{{2t}}\left\| {{\textit z} - x} \right\|_2^2 + h\left( {\textit z} \right)\\
=\;& argmi{n_{\textit z}}\;\frac{1}{{2t}}\left\| {{\textit z} - (x - t\nabla g\left( x \right)} \right\|_2^2 + h\left( {\textit z} \right)
\end{split}\end{equation*}

The operator $  {Prox}_{t,h}\left(x\right)={argmin}_{{\textit z}}\dfrac{1}{2t}{\left|\right|{\textit z}-x\left|\right|}_{2}^{2}+h\left({\textit z}\right) $ is called proximal mapping for $ h $. To solve (7), the key is to compute the proximal mapping for the Berhu function:

$  \lambda {B}_{M}\left({\textit z}\right)=\lambda \left|{\textit z}\right|{1}_{\left|{\textit z}\right|\le M}+\lambda \frac{{{\textit z}}^{2}+{M}^{2}}{2M}{1}_{\left|{\textit z}\right|>M}=\lambda \left|{\textit z}\right|+\lambda \frac{{\left(\right|{\textit z}|-M)}^{2}}{2M}{1}_{\left|{\textit z}\right|>M} $

let $u\left({\textit z}\right)=\lambda \frac{{\left(\right|{\textit z}|-M)}^{2}}{2M}{1}_{\left|{\textit z}\right| > M}$. As $  u\left({\textit z}\right) $ satisfies theorem 4 in^[46]^:

8 $
\begin{array}{c}{Prox}_{t,\lambda B}\left(x\right)={Prox}_{t,\lambda u}\left(x\right)\circ {Prox}_{t,\lambda \left|\cdot \right|}\left(x\right)\end{array} 
$

It is not difficult to verify:

9 $
{Prox}_{t,\lambda u}\left(x\right)=sign\left(x\right)\mathrm{min}\left\{\left|x\right|,\frac{M}{M+t\lambda }\left(\left|x\right|+t\lambda \right)\right\}
$

10 $
{Prox}_{t,\lambda \left|\cdot \right|}\left(x\right)=sign\left(x\right)\mathrm{min}\left\{\left|x\right|-t\lambda ,0\right\}
$

Finding $  {\beta }_{0} $ and ${\boldsymbol{\beta }}$ that minimize $  f\left(\,{\boldsymbol{\beta }}\right) $ in (7) is detailed in Algorithm 1.

**Table 1:** 

**Algorithm 1:** Accelerated proximal gradient descent method to minimize $ f\left({\boldsymbol{\beta }}\right) $ in equation (7) respected to $ {\beta }_{0} $ and ${\boldsymbol{\beta}}$	
Input: predictor matrix ($X $), dependent vector ($y $), and penalty constant ($ {\boldsymbol{\lambda}}$)	
Output: regression coefficient ($ {\boldsymbol{\beta }} $)	
1	Initiate $ {\boldsymbol{\beta }}={\bf{0}} $, $\boldsymbol{t}$ **= 1**, $ {{\boldsymbol{\beta }}}_{\boldsymbol{p}\boldsymbol{r}\boldsymbol{e}\boldsymbol{v}}={\bf{0}} $
2	For $k $ in 1… MAX_ITER
3	$v={\boldsymbol{\beta }}+\left(k/\left( {k + 3} \right)\right)\boldsymbol{*}\left({\boldsymbol{\beta }}-{{\boldsymbol{\beta }}}_{\boldsymbol{p}\boldsymbol{r}\boldsymbol{e}\boldsymbol{v}}\right)$
4	compute the gradient of Huber loss at $ v $ using (5), denoted as $ {\boldsymbol{G}}_{\boldsymbol{v}} $
5	while TRUE
6	compute $ {\boldsymbol{p}}_{1}={\boldsymbol{P}\boldsymbol{r}\boldsymbol{o}\boldsymbol{x}}_{\boldsymbol{t},\boldsymbol{\lambda }\left|\cdot \right|}\left(\boldsymbol{v}\right) $ using (10)
7	compute ${\boldsymbol{p}}_{2}={\boldsymbol{P}\boldsymbol{r}\boldsymbol{o}\boldsymbol{x}}_{\boldsymbol{t},\boldsymbol{\lambda }\boldsymbol{u}}\left(\boldsymbol{p}_1\right)$ using (9)
8	if ${\bf\sum }_{i=1}^{n}{\boldsymbol{H}}_{\boldsymbol{M}}\left({\boldsymbol{y}}_{\boldsymbol{i}} -{\boldsymbol{\beta}}_{\boldsymbol{0}}- {\boldsymbol{x}}_{\boldsymbol{i}}^{\boldsymbol{T}}{\boldsymbol{p}}_{2}\right)\le {\sum }_{i=1}^{n}{\boldsymbol{H}}_{\boldsymbol{M}}\left({\boldsymbol{y}}_{\boldsymbol{i}} -{\boldsymbol{\beta}}_{\boldsymbol{0}}- {\boldsymbol{x}}_{\boldsymbol{i}}^{\boldsymbol{T}}\boldsymbol{v}\right)+$${\boldsymbol{G}}_{\boldsymbol{v}}'({\boldsymbol{p}}_{\bf 2} -\boldsymbol{v})+ \frac{\bf 1}{\bf 2\boldsymbol{t}}{\left|\right|{\boldsymbol{p}}_{\bf 2}-\boldsymbol{v}\left|\right|}_{\bf 2}^{\bf 2}$
9	break
10	else $ t=t*0.5 $
11	$ {{\boldsymbol{\beta }}}_{\boldsymbol{p}\boldsymbol{r}\boldsymbol{e}\boldsymbol{v}}={\boldsymbol{\beta }} $, $ {\boldsymbol{\beta }}={\boldsymbol{p}}_{2} $
12	if converged
13	break

Algorithm 1 uses the accelerated proximal gradient descent method to solve (7). Line 3 implements the acceleration of^[47]^. Lines 6−7 compute the proximal mapping of the Berhu function. Lines 5−10 use a backtracking method to determine the step size.

### Embedding the Huber-Berhu objective function into PLS

Let $  X (n\times p) $ and $  Y (n\times q) $ be the standardized predictor variables (gene expression of TF genes) and dependent variables (gene expression of pathway genes), respectively. PLS^[48]^ looks for a linear combination of $ X $ and a linear combination of $ Y $ such that their covariance reaches a maximum:

11 $
{ma{x_{{{\left\| u \right\|}_2} = 1,{{\left\| v \right\|}_2} = 1}}cov\left( {Xu,Yv} \right)}
$

Here, the linear combination $  {\xi} =Xu $ and $  \eta =Yv $ are called component scores (or latent variables) which are generated through the $ p $ and $ q $ dimensional weight vectors $ u $ and $ v $, respectively. After getting this first component $ {\xi}  $, two regression equations (from $ X $ to $ {\xi}  $ and from $ Y $ to $ {\xi}  $) were set up:

12 $
X={\xi} {c}'+{\varepsilon }_{1}, Y={\xi} {d}'+{\varepsilon }_{2}=Xb+{\varepsilon }_{3}
$

Here, $ c $ and $ d $ are commonly called loadings in the literature. Next, $ X $ was deflated as $  X=X-{\xi} {c}' $ and $ Y $ was deflated as $  Y=Y-{\xi} {d}' $, and this process was continued until enough components were extracted.

A close relationship exists between PLS and SVD. Let $  M=X'Y $, then $  cov\left(Xu,Yv\right)=\dfrac{1}{n}u'Mv $. Let the SVD of $ M $ be:

$  M=U\Delta V' $

where $  U(p\times r) $ and $  V(q\times r) $ are orthonormal and $  \Delta (r\times r) $ is a diagonal matrix whose diagonal elements $  {\delta }_{k}(k=1\dots r) $ are called singular values. According to the property of SVD, the combinatory coefficients $ u $ and $ v $ in (7) are exactly the first column of $ U $ and the first column of $ V $. Therefore, the weight vectors of PLS can be computed by:

$mi{n_{u,v}}\left\| {M - uv'} \right\|_F^p$

where $\left\| {M - uv'} \right\|_F^p = \mathop \sum \nolimits_{i = 1}^p \mathop \sum \nolimits_{j = 1}^q {\left( {{m_{ij}} - {u_i}{v_j}} \right)^2}$.

Lê Cao et al. 2008^[38]^ proposed a sparse PLS approach using SVD decomposition of $ M $ by adding a $ {\ell}_{1} $ penalty on the weight vectors. The optimization problem to solve is:

$mi{n_{u,v}}\left\| {M - uv'} \right\|_F^p + {\lambda _1}{\left\| u \right\|_1} + {\lambda _2}{\left\| v \right\|_1}$

As mentioned above, the Huber function is more robust to outliers and has higher statistical efficiency than LAD loss, and the Berhu penalty has a better balance between the $ {\ell}_{1} $ and $ {\ell}_{2} $ penalty. The Huber loss and the Berhu penalty were adopted to extract each component for the PLS regression. The optimization problem becomes:

13 $
{min}_{u,v} {\sum }_{i=1}^{p}{\sum }_{j=1}^{q}H\left({m}_{ij}-{u}_{i}{v}_{j}\right)+\lambda {\sum }_{i=1}^{p}B\left({u}_{i}\right)+\lambda {\sum }_{i=1}^{q}B\left({v}_{i}\right)
$

The objective function in (13) is not convex on $ u $ and $ v $, but it is convex on $ u $ when $ v $ is fixed and convex on $ v $ when $ u $ is fixed. For example, when $ v $ is fixed, each $ {u}_{i} $ in parallel can be solved by:

14 $
{min}_{{u}_{i}}{\sum }_{j=1}^{q}H\left({m}_{ij}-{u}_{i}{v}_{j}\right) +\lambda B\left({u}_{i}\right)
$

Similarly, when $ u $ is fixed, each $ {v}_{j} $ in parallel can be computed by:

15 $
{min}_{{v}_{j}} {\sum }_{i=1}^{p}H\left({m}_{ij}-{u}_{i}{v}_{j}\right)+\lambda B\left({v}_{j}\right) 
$

Equations (14) and (15) can be solved using Algorithm 1. Therefore (13) can be solved iteratively by updating $ u $ and $ v $ alternately. Note, it is not cost-efficient to spend a lot of effort optimizing over $ u $ in line 6 before a good estimate for $ v $ is computed. Since Algorithm 1 is an iterative algorithm, it may make sense to stop the optimization over $ u $ early before updating $ v $. In the implementation, one step of proximal mapping was used to update $ u $ and $ v $. That is:

16 $
u={Prox}_{t,\lambda B}\left(u-t\frac{\partial H\left(M-u{v}'\right)}{\partial u}\right)
$

17 $
v={Prox}_{t,\lambda B}\left(v-t\frac{\partial H\left(M-u{v}'\right)}{\partial v}\right)
$

The algorithm for finding the solution of the Huber–Berhu PLS regression in (13) is detailed in Algorithm 2.

**Table 2:** 

**Algorithm 2:** Finding the solution of the Huber-Berhu PLS regression	
Input: TF matrix ($ X $), pathway matrix ($ Y $), penalty constant (${\boldsymbol{\lambda}}$), and number of components ($ K $)	
Output: regression coefficient matrix ($ A $)	
1	$ {\boldsymbol{X}}_{0}=\boldsymbol{X},{\boldsymbol{X}}_{0}={\boldsymbol{Y}} $, $ {\boldsymbol{c}\boldsymbol{F}}={\boldsymbol{I}} $, $ {\boldsymbol{A}}={\bf{0}} $
2	For $ k $ in 1,...,$K $
3	set $ {\boldsymbol{M}}_{\boldsymbol{k}-1}={\boldsymbol{X}}_{\boldsymbol{k}-1}'{\boldsymbol{Y}}_{\boldsymbol{k}-1} $
4	Initialize $ {\boldsymbol{u} }$ to be the first left singular vector and initialize $ {\boldsymbol{v}} $ to be the product of first right singular vectors and first singular value.
5	until convergence of $ {\boldsymbol{u}} $ and $ {\boldsymbol{v}} $
6	update ${ \boldsymbol{u}} $ using (16)
7	update $ {\boldsymbol{v}} $ using (17)
8	extract component $ {{{\xi}}} ={\boldsymbol{X}\boldsymbol{u}} $
9	compute regression coefficients in (8) ${\boldsymbol c}={\boldsymbol X}'{{{\xi}}}/({{{\xi}}}'{{{\xi}}}), \;{\boldsymbol d}={\boldsymbol Y}'{{{\xi}}}/$$({{{\xi}}}'{{{\xi}}}) $
10	update $\boldsymbol{A}=\boldsymbol{A}+\boldsymbol{c}\boldsymbol{F}\cdot \boldsymbol{u}\cdot \boldsymbol{d}'$
11	update ${\boldsymbol{c}\boldsymbol{F}}={\boldsymbol{c}\boldsymbol{F}}\cdot ({\bf{I}}-{\boldsymbol{u}}\cdot {\boldsymbol{c}}'$)
12	compute residuals for $X $ and $ Y$, ${\boldsymbol{X}}={\boldsymbol{X}}- {{{\xi}}}{\boldsymbol c}'$, $ {\boldsymbol{Y}}= {\boldsymbol{Y}}- {{{\xi}}}{\boldsymbol d}$

### Tuning criteria and choice of the PLS dimension

The Huber-Berhu PLS regression has two tuning parameters, namely, the penalization parameter $ \lambda  $ and the number of hidden components $ K $. To select the best penalization parameter, $ \lambda  $, a common $k $-fold cross-validation (CV) procedure that minimizes the overall prediction error is applied using a grid of possible values. If the sample size is too small, CV can be replaced by leave-one-out validation; this procedure is also used in for tuning penalization parameters^[37,49]^.

To choose the dimension of PLS, the $ {Q}_{h}^{2} $ criteria were adopted. $ {Q}_{h}^{2} $ criteria were first proposed by Tenenhaus^[50]^. These criteria characterize the predictive power of the PLS model by performing cross-validation computation. $ {Q}_{h}^{2} $ is defined as:

$  {Q}_{h}^{2}=1-\frac{{\sum }_{k=1}^{q}{PRESS}_{h}^{k}}{{\sum }_{k=1}^{q}{RSS}_{h}^{k}} $

where $  {PRESS}_{h}^{k}={\sum }_{i=1}^{n}{({y}_{i}^{k}-{\hat{y}}_{h(-i)}^{k})}^{2} $ is the Prediction Error Sum of Squares, and $  {RSS}_{h}^{k}={\sum }_{i=1}^{n}{({y}_{i}^{k}-{\hat{y}}_{h}^{k})}^{2} $ is the Residual Sum of Squares for the variable $ k $ and the PLS dimension $ h $. The criterion for determining if $ {{\xi} }_{h} $ contributes significantly to the prediction is:

$  {Q}_{h}^{2}\ge \left(1-{0.95}^{2}\right)=0.0975 $

This criterion is also used in SIMCA-P software^[51]^ and sparse PLS^[38]^. However, the choice of the PLS dimension still remains an open question. Empirically, there is little biological meaning when $ h $ is large and good performance appears in 2−5 dimensions.

## RESULTS

### The efficiency of the proximal gradient descent algorithm

**Figure 3:**
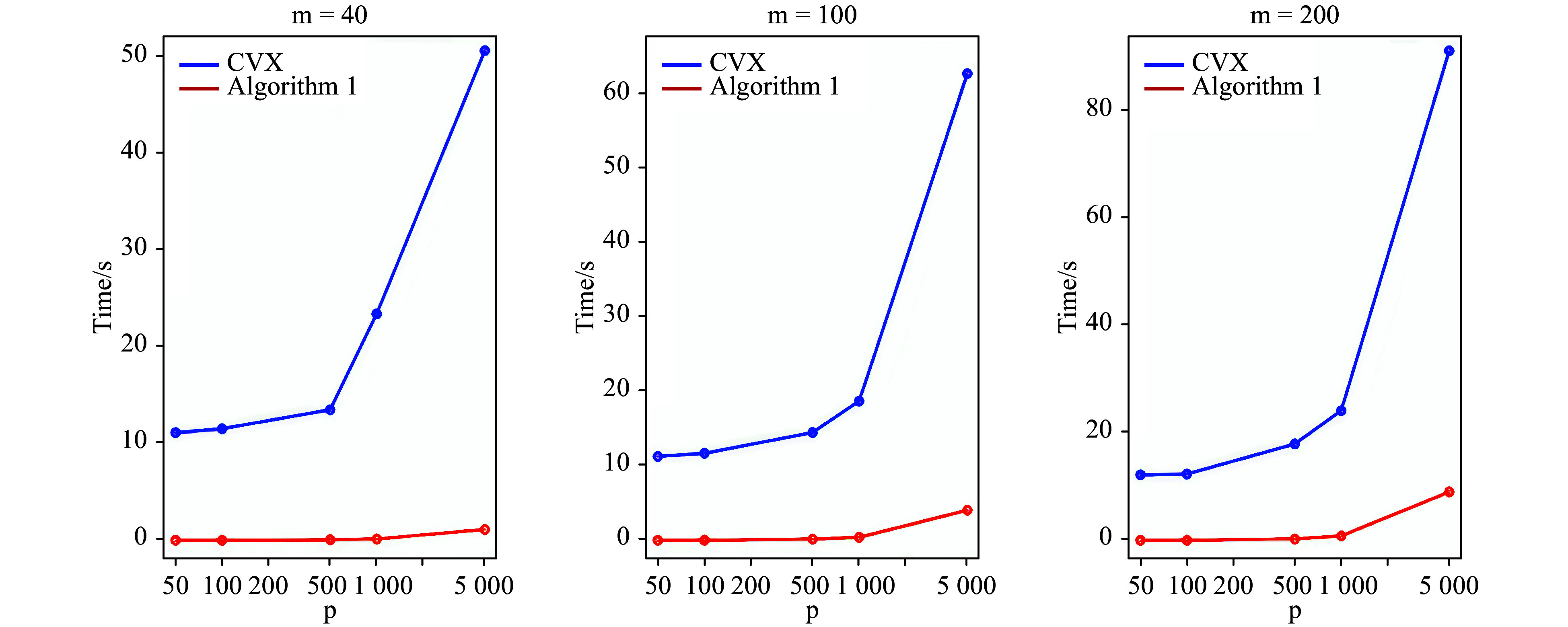
Comparison of running time for Algorithm 1 and CVX. $p $ is the number of independent variables in TF-matrix ($X $).

We developed the proximal gradient descent algorithm (Algorithm 1) to solve Huber-Berhu regression. As compared to CVX, it could reduce the running time to at least 10 times, but up to 90 times in a desktop computer with 2.2 GHz Intel Core i7 processor and 16 GB 1600 MHz DDR3 memory for a setting of $m $ and $p $ based on 30 replications. For different $m $, the patterns are similar (Fig. 3). More details can be found in the Deng 2018^[52]^.

### Validation of Huber-Berhu PLS with lignin biosynthesis pathway genes and regulators

**Figure 4:**
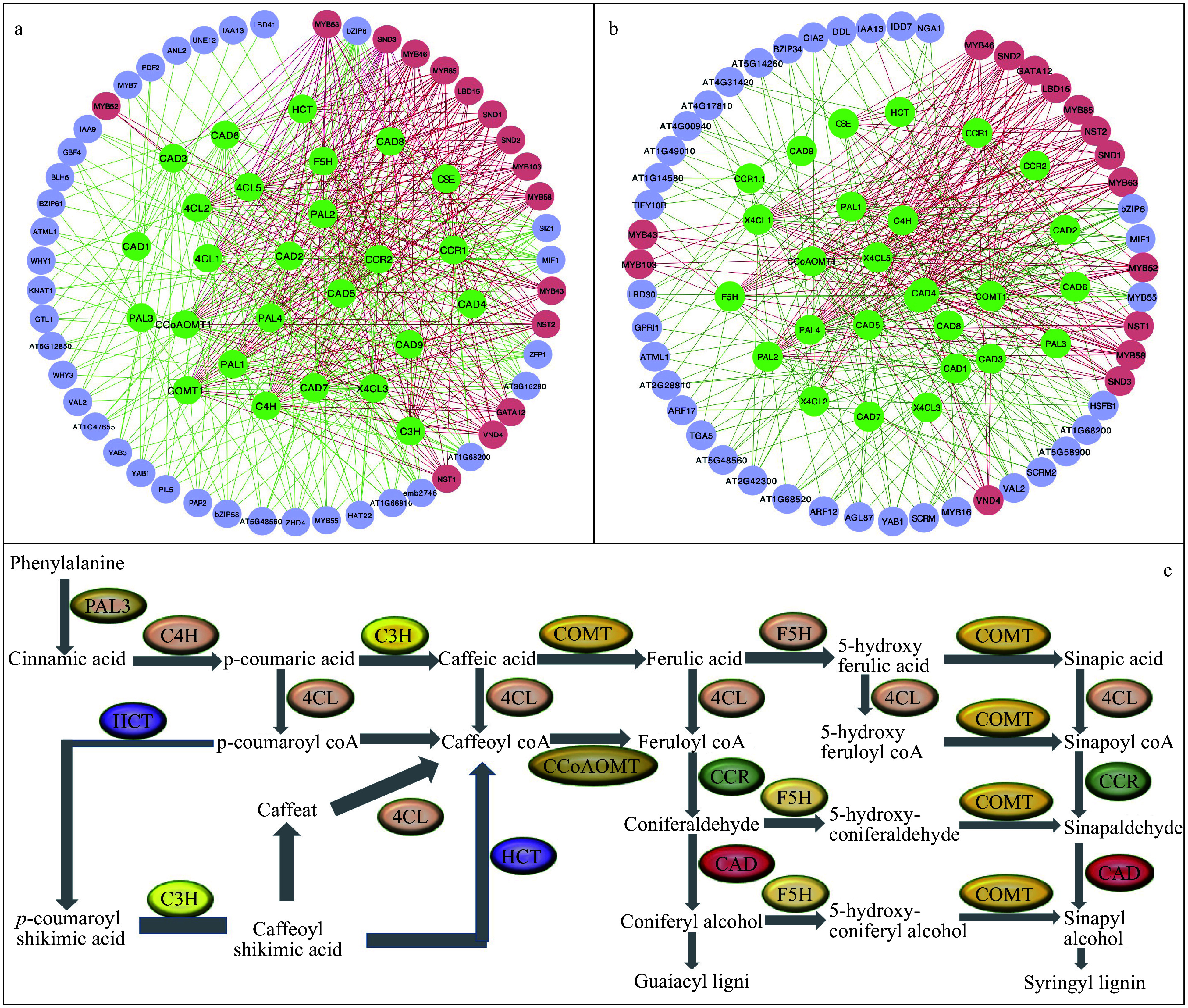
The implementation of Huber-Berhu-Partial Least Squares (HB-PLS) to identify candidate regulatory genes controlling lignin biosynthesis pathway. (a) HB-PLS; (b) SPLS. Green nodes (inside the circles) represent lignin biosynthesis genes. Coral nodes represent positive lignin pathway regulators supported by existing literature, and shallow purple nodes contain other predicted transcription factors that are not supported by current available literature. (c) The lignin biosynthesis pathway.

The HB-PLS algorithm was examined for its accuracy in identifying lignin pathway regulators using the *A. thaliana* microarray compendium data set produced from stem tissues^[40]^. TFs identified by HB-PLS were compared to those identified by SPLS. The 50 top TFs that were ranked based on their connectivities with the lignin biosynthesis pathway genes were identified using HB-PLS (Fig. 4a) and compared to those identified by SPLS (Fig. 4b), respectively. The lignin biosynthesis pathway genes are shown in Fig. 4c. The positive lignin biosynthesis pathway regulators, which are supported by literature evidence, are shown in coral color. The HB-PLS algorithm identified 15 known lignin pathway regulators. Of these, MYB63, SND3, MYB46, MYB85, LBD15, SND1, SND2, MYB103, MYB58, MYB43, NST2, GATA12, VND4, NST1, MYB52, are positive known transcriptional activators of lignin biosynthesis in the SND1-mediated transcriptional regulatory network^[53]^, and LBD15^[54]^ and GATA12^[55]^ are also involved in regulating various aspects of secondary cell wall synthesis. Interestingly, SPLS identified the same set of positive pathway regulators as HB-PLS though their ranking orders are different.

### Prediction of photosynthetic pathway regulators in *Arabidopsis thaliana* using Huber-Berhu PLS

**Figure 5:**
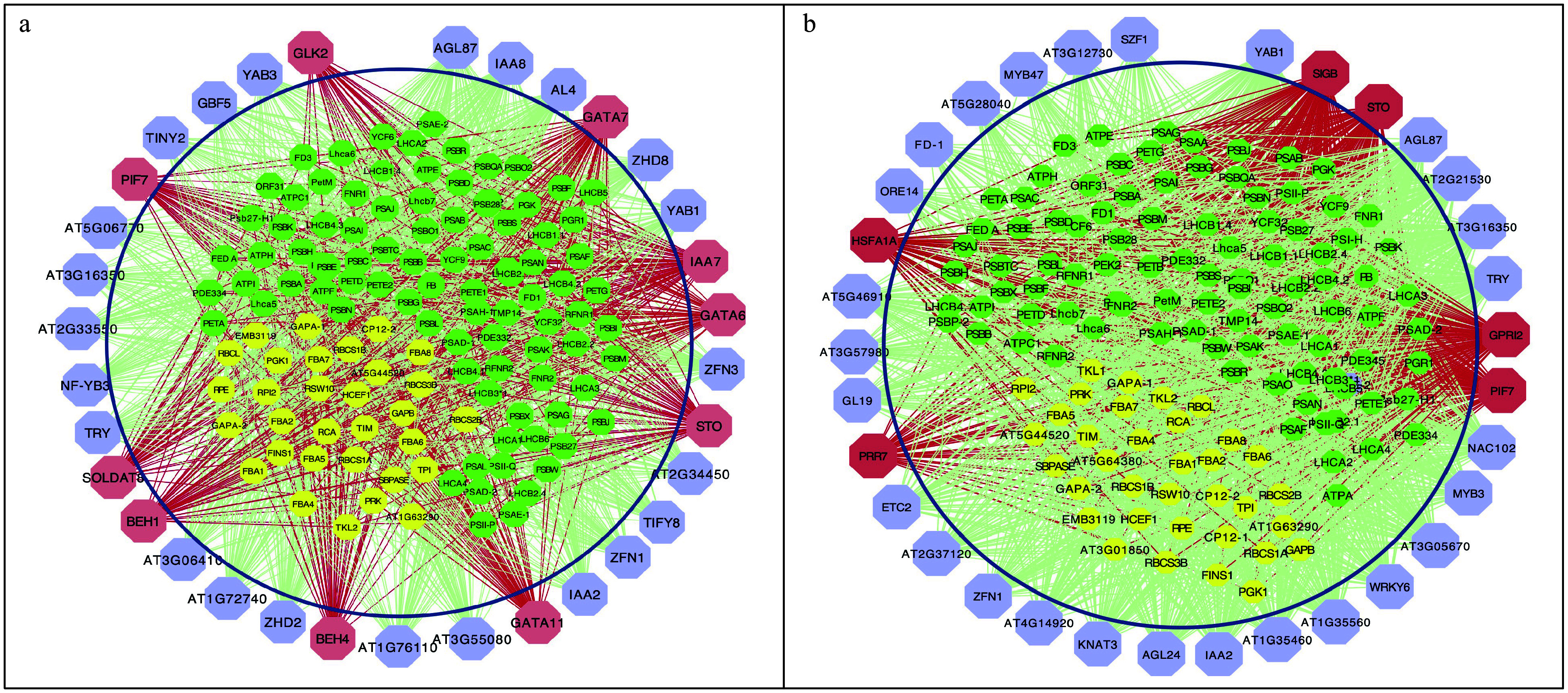
The implementation of Huber-Berhu-Partial Least Squares (HB-PLS) to identify candidate regulatory genes (purple and coral nodes) controlling photosynthesis and related pathway genes. (a) was compared with the sparse partial least squares (SPLS) method (b) in identifying regulators that affects maize photosynthesis light reaction and Calvin cycle pathway genes. The green and yellow nodes within the cycles represent photosynthesis light reaction pathway genes and Calvin cycle pathway genes, respectively. Coral nodes in the circles represent positive predicted biological process or pathway regulators that are supported by existing literature, and shallow purple nodes contain other predicted TFs that do not have experimentally validated supporting evidence at present.

Photosynthesis is mediated by the coordinated action of approximately 3,000 different proteins, commonly referred to as photosynthesis proteins^[56]^. In this study, we used genes from the photosynthesis light reaction pathway and Calvin cycle pathway to study which regulatory genes can potentially control photosynthesis. Analysis was performed using HB-PLS, with SPLS as a comparative method. The compendium data set we used is comprised of 238 RNA-seq data sets from *Arabidopsis thaliana* leaves that were under normal/untreated conditions. Expression data for 1389 TFs and 130 pathway genes were extracted from the above compendium data set and used for analyses. The results of HB-PLS and SPLS methods are shown in Fig. 5a and 5b, respectively, where 33 rather than 50 TFs were shown because the SPLS method only identified 33 TFs. Of the top 33 candidate TFs in the lists, HB-PLS identified 11 positive known TFs while SPLS identified 6 positive known TFs. *IAA7*, also known as *AXR2*, is regulated by HY5^[57]^, which binds to G-box in LIGHT-HARVESTING CHLOROPHYLL A/B (Lhcb) proteins^[58]^. *STO*, also known as *BBX24*, whose protein physically interacts with photosynthesis regulator HY5 to control photomorphogenesis^[59]^; PHYTOCHROME-INTERACTING FACTOR (PIF) family have been shown to affect the expression of photosynthesis-related genes, including genes encoding LHCA, LHCB, and PsaD proteins^[60−62]^. PIFs repress chloroplast development and photomorphogenesis^[62]^; PIF7, together with PIF3 and PIF4, regulates responses to prolonged red light by modulating phyB levels^[63]^. PIF7 is also involved in the regulation of circadian rhythms. GLK2, directly regulate the expression of a series of photosynthetic genes including the genes encoding the PSI-LHCI complex and PSII-LHCII complex^[64,65]^. The plastid sigma-like transcription factor SIG1 regulate *psaA* respectively^[66]^; TOC1 is a member of the PRR (PSEUDO-RESPONSE REGULATOR) family that includes PRR9, PRR7, PRR5, PRR3, and PRR1/TOC1. HY5 also binds and regulates the circadian clock gene *PRR7*, which affects the operating efficiency of PSII under blue light^[67]^. GATA transcription factors have implicated some proteins in light-mediated and circadian-regulated gene expression^[68,69]^, GATAs can bind to XXIII box, a cis-acting elements involved in light-regulated expression of the nuclear gene GAPB, which encodes the B subunit of chloroplast glyceraldehyde-3-phosphate dehydrogenase in *A. thaliana*^[70]^. In addition, GATA interacts with SORLIP motifs in the 3-hydroxy-3-methylglutaryl-CoA reductase (*HMGR*) promoter of *Picrorhiza kurrooa*, a herb plant, for the control of light-mediated expression; upstream sequences of *HMGR* of *P. kurrooa* (*PropkHMGR*)-mediated gene expression was higher in the dark as compared to that in the light in *A. thaliana* across four temperatures studied^[71]^. GATA phytochrome interacting factor transcription factors regulate light-induced vindoline biosynthesis in *Catharanthus roseus*^[72]^. A number of genes show greater than 2-fold higher expression in light-grown than dark-grown seedlings with the greatest differences observed for *GATA6*, *GATA7*, *GATA21*-23^[68]^, with *GATA6 and 7* showing about 6- and 4-fold difference in expression levels. GATA11 is found to be a hub regulator of photosynthesis and Chlorophyll biosynthesis^[73]^. The GLK transcription factors promote the expression of many nuclear-encoded photosynthetic genes that are associated with chlorophyll biosynthesis and light-harvesting functions^[74]^; HSFA1, a master regulator of transcriptional regulation under heat stress, regulates photosynthesis by inducing the expression of downstream transcription factors^[75]^. *BEH1* is a homolog of *BZR1*, genetic analysis indicates that the BZR1-PIF4 interaction controls a core transcription network by integrating brassinosteroids and light response^[76]^.

### The performance and sensitivity of HB-PLS using SPLS as a comparison

**Figure 6:**
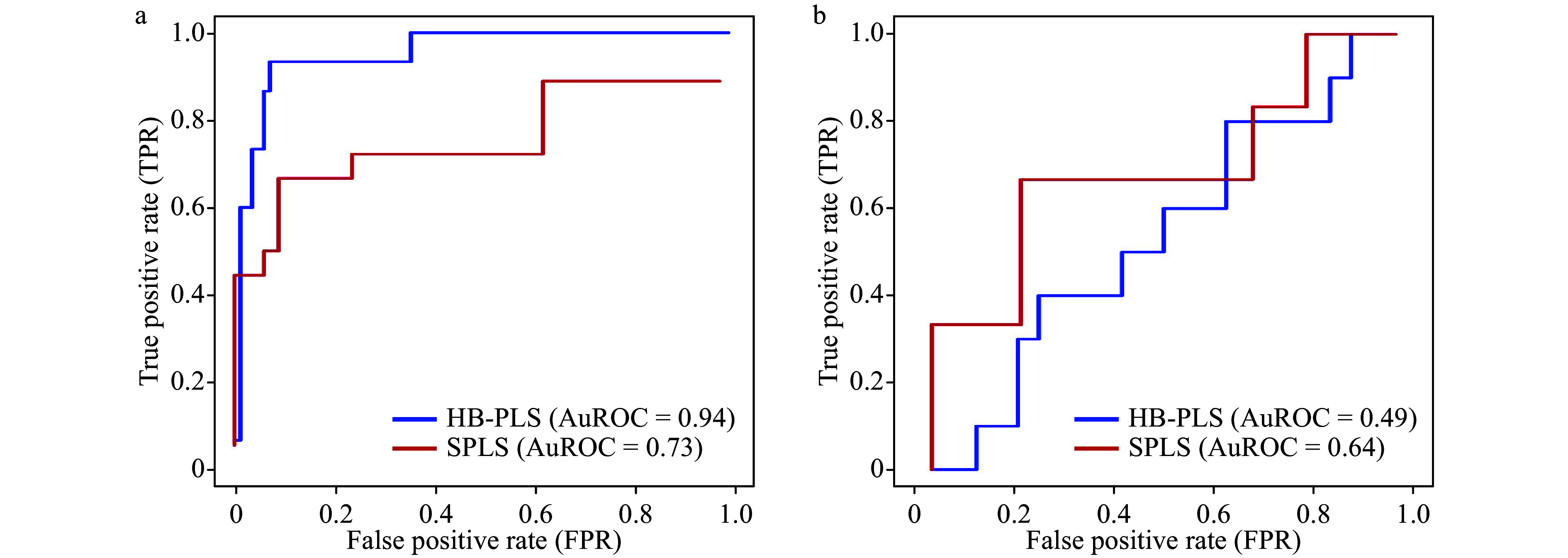
The receiver operating characteristic (ROC) curves of Huber-Berhu-partial least squares (HB-PLS) and sparse partial least squares (SPLS) methods for identifying pathway regulators in *Arabidopsis thaliana*. (a) Lignin biosynthesis pathway; (b) a merged pathway of light reaction pathway and Calvin cycle pathway.

We tested the HB-PLS method in comparison with SPLS using two metabolic pathways, lignin biosynthesis pathway and a unified photosynthesis pathway whose regulatory genes are largely and partially known, respectively. We found that HB-PLS could identify more positive known TFs that are supported by existing literature in the output lists. To examine which methods can rank relatively more positive known TFs to the top of output regulatory gene lists, we plotted receiver operating characteristic curves (ROC) and calculated the area under the ROC curve (AuROC), which reflects the sensitivity versus 1-specificity of a method. The results are shown in Fig. 6. For lignin biosynthesis pathway, HB-PLS was capable of ranking more positive known pathway regulators to the top in the inferred regulatory gene list. As a result, the AuROC of HB-PLS (0.94) (Fig. 6a) is much large than that of SPLS (0.73) (Fig. 6b). For the unified light reaction and Calvin cycle pathway, the true pathway regulators have not been fully identified, and they are only partially known. Although SPLS only identified the 6 positive known pathway regulators in comparison with 10 identified by HB-PLS, SPLS ranked 4 out 6 positive known pathway regulators to the top 8 positions, resulting in slightly higher sensitivity versus 1-specificity. HB-PLS identified 10 positive known regulators among the top 33 regulatory genes, which are more evenly distributed in the list, resulting in relatively smaller AuROC (0.49) as compared to the AuROC of SPLS (0.64). The overall lower AuROC values for both methods for photosynthesis pathway are probably owing to the low number of positive known regulatory genes for this pathway.

Given the fact that lignin biosynthesis pathway regulators have been well identified and characterized experimentally^[77]^, they are specifically suited for examining the efficiency of the HB-PLS method for each pathway gene. We selected two methods, SPLS and PLS, as comparisons. For each output TF list to a pathway gene yielded from one of three methods, we applied a series of cutoffs, with the number of TFs retained varying from 1 to 40 in a shifting step of 1 at a time, and then counted the number of positive regulatory genes in each of the retained lists. The results are shown in Supplementary Fig. S1. It is obvious that for almost every pathway gene, HB-PLS has higher sensitivity versus specificity.

The results indicate that the HB-PLS and SPLS regressions, in many cases, are much more efficient in recognizing positive regulators to a pathway gene compared to the PLS regression (Supplementary Fig. S1). For most pathway genes like *PAL1*, *C4H*, *CCR1*, *C3H*, and *COMT1*, HB-PLS method could identify more positive regulators in the top 20 regulators as compared to the SPLS method. For *HCT*, *CCoAOMT1*, *CAD8*, and F5H, HB-PLS was almost always more efficient than SPLS when the top cut-off lists contained fewer than 40 regulators. For pathway gene *CAD8*, both SPLS and PLS both failed to identify positive regulators while HB-PLS performed more efficiently.

## DISCUSSION

**Figure 7:**
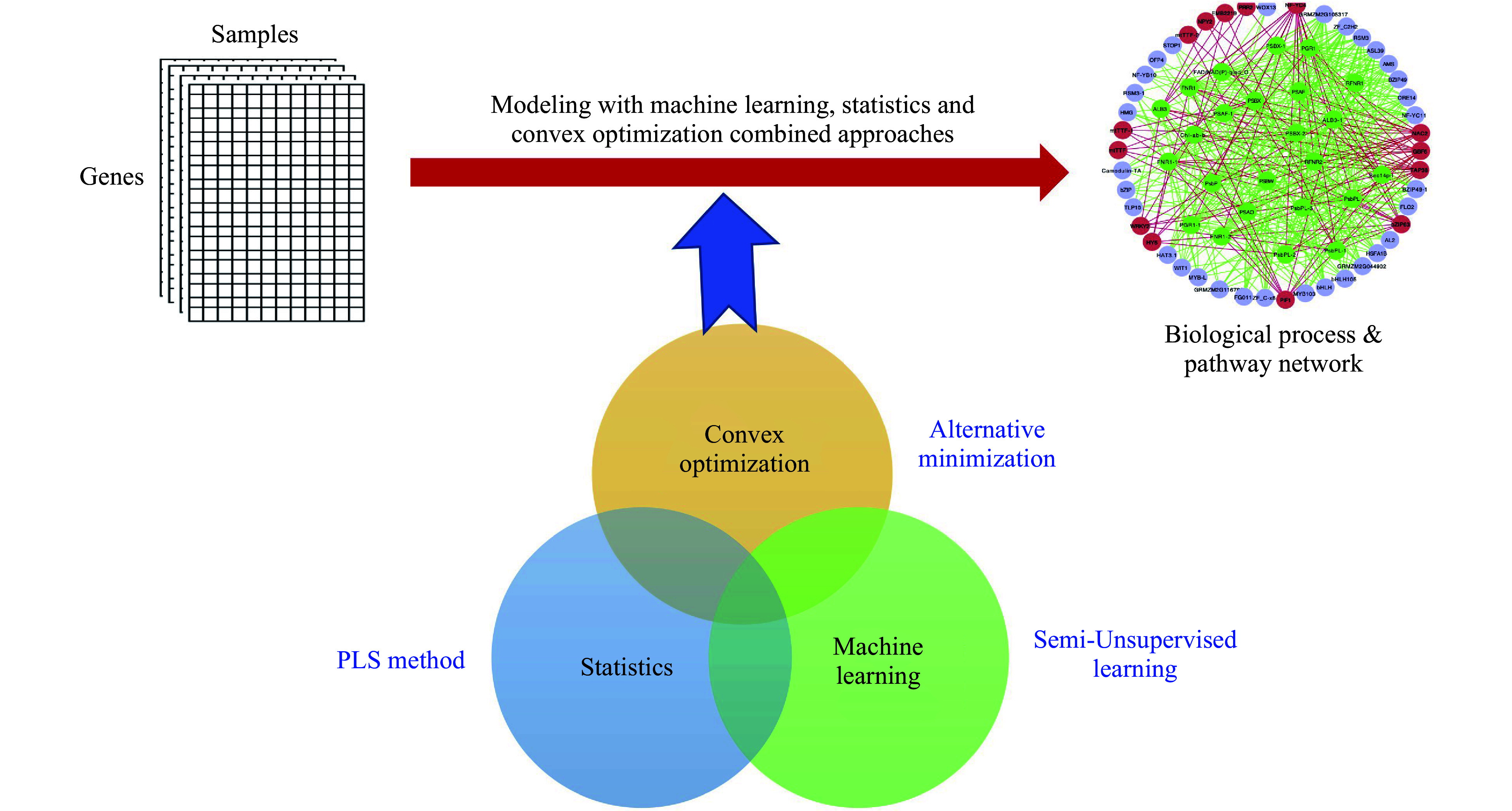
An integrative framework for identifying biological process and pathway regulators from high-throughput gene expression data by integration of statistics, machine learning and convex optimization. PLS: Partial least squares.

The identification of gene regulatory relationships through constructing GRNs from high-throughput expression data sets has some inherent challenges due to high dimensionality and multicollinearity. High dimensionality is caused by a multitude of gene variables while multicollinearity largely results from a large number of genes versus a relatively small sample size. In this study, we combined three types of computational approaches, statistics (PLS), machine learning (Semi-unsupervised learning) and convex optimization (Berhu and Huber) for simulating gene regulatory relationships, as illustrated in Fig. 7, and our results showed this integrative approach is viable and efficient.

One method that we frequently use to deal with dimensionality and multicollinearity is partial least squares (PLS), which couples dimension reduction with a regression model. However, because PLS is not particularly suited for variable/feature selection, it often produces linear combinations of the original predictors that are hard to interpret due to high dimensionality^[78]^. To solve this problem, Chun and Keles developed an efficient implementation of sparse PLS, referred to as the SPLS method, based on the least angle regression^[79]^. SPLS was then benchmarked by means of comparisons to well-known variable selection and dimension reduction approaches via simulation experiments^[78]^. We used the SPLS method in our previous study^[41]^ and found that it was highly efficient in identifying pathway regulators and thus used it as a benchmark for evaluating the new methods.

In this study, we developed a PLS regression that incorporates the Huber loss function and the Berhu penalty for identification of pathway regulators using high-throughput gene expression data (with dimensionality and multicollinearity). Although the Huber loss function and the Berhu penalty have been proposed in regularized regression models^[43,80]^, this is the first time that both of them were combined with the PLS regression at the same time. The Huber function is a combination of linear and quadratic loss functions. In comparison with other loss functions (e.g., square loss and least absolute deviation loss), Huber loss is more robust to outliers and has higher statistical efficiency than the LAD loss function in the absence of outliers. The Berhu function^[33]^ is a hybrid of the $ {\ell}_{2} $ penalty and the $ {\ell}_{1} $ penalty. It gives a quadratic penalty to large coefficients and a linear penalty to small coefficients. Therefore, the Berhu penalty has advantages of both the $ {\ell}_{2} $ and $ {\ell}_{1} $ penalties: smaller coefficients tend to shrink to zero while the coefficients of a group of highly correlated predictive variables are not changed much if their coefficients are large.

A comparison of HB-PLS with SPLS and also PLS suggests that HB-PLS can identify more true pathway regulators. This is an advantage over either SPLS or PLS (Supplementary Fig. S1) when experimental validation is concerned. The application of HB-PLS and SPLS methods to identification of lignin biosynthesis pathway regulators in *A. thalian* led to the identification of 15 and 15 positive pathway regulators, respectively, while application of the HB-PLS and SPLS methods to identification of photosynthesis pathway regulators in *A. thalian* resulted in 10 and 6 positive pathway regulators, respectively. The outperformance of HB-PLS over SPLS (Fig. 6a) and PLS (Supplementary Fig. S1) implicates that the use of Huber loss function and Berhu penalty function for convex optimization contributed to the recognition of true pathway regulators and rank them at the top of the output lists. It also suggests the viability and the increased power of combination of statistics (PLS), machine learning (Semi-unsupervised learning) and convex optimization (Berhu and Huber) for recognition of regulatory relationships. In addition, the ROC plotting suggests that HB-PLS method has comparable sensitivity versus 1-specificity compared to SPLS because HB-PLS achieved a higher AuROC for lignin biosynthesis pathway but a lower AuROC for the unified photosynthesis pathway as compared to SPLS (Fig. 6). However, the fact that the HB-PLS identified the same or higher number of positive true regulators than SPLS for the two pathways we analyzed, and the sensitivity of HB-PLS is much better than that of SPLS for lignin pathway whose regulatory genes are more complete, and slightly worse than that of HB-PLS for photosynthesis light reaction and Calvin cycle pathway (Fig. 5 and Supplementary Fig. S1) whose regulatory genes are only partially known. Therefore, HB-PLS has an overall larger advantage. Unfortunately, except the two pathways we evaluated, there are almost no other metabolic pathways whose regulatory genes have been mostly identified. Our analysis showed that the two methods could empower the recognition of pathway regulators including some unique pathway regulators, and thus are useful in continued research.

## CONCLUSIONS

A new method called the HB-PLS regression was developed for identifying biological process or pathway regulators by integration of statistics, machine learning and convex optimization approaches. In HB-PLS, an accelerated proximal gradient descent algorithm was specifically developed to solve Huber and Berhu optimization, which can estimate the regression parameters by optimizing the objective function based on the Huber and Berhu functions. Characteristic analysis of the Huber-Berhu regression indicated it could identify sparse solution. When modeling the gene regulatory relationships from regulatory genes to pathway genes, HB-PLS is capable of dealing with the high multicollinearity of both regulatory genes and pathway genes. Application of the HB-PLS to real *A. thaliana* high-throughput data showed that HB-PLS could identify majority positive known regulatory genes that govern two pathways. Sensitivity verse 1-specificity plotting showed that HB-PLS could rank more positive known regulators to the top of output regulatory gene lists for lignin biosynthesis pathways while SPLS can rank more for the unified photosynthesis pathway. Our study suggests that the overall performance of HB-PLS exceeds that of SPLS but both methods may have comparable sensitivity/specificity and are instrumental for identifying real biological process and pathway regulators from high-throughput gene expression data.

## SUPPLEMENTARY DATA

Supplementary data to this article can be found online.
