# Supplementary figures and images for: HB-PLS: A statistical method for identifying biological process or pathway regulators by integrating Huber loss and Berhu penalty with partial least squares regression
Source: For Res (Fayettev). 2021 Mar 30;1:6. doi: 10.48130/FR-2021-0006 (PMC11524267; doi:10.48130/FR-2021-0006)

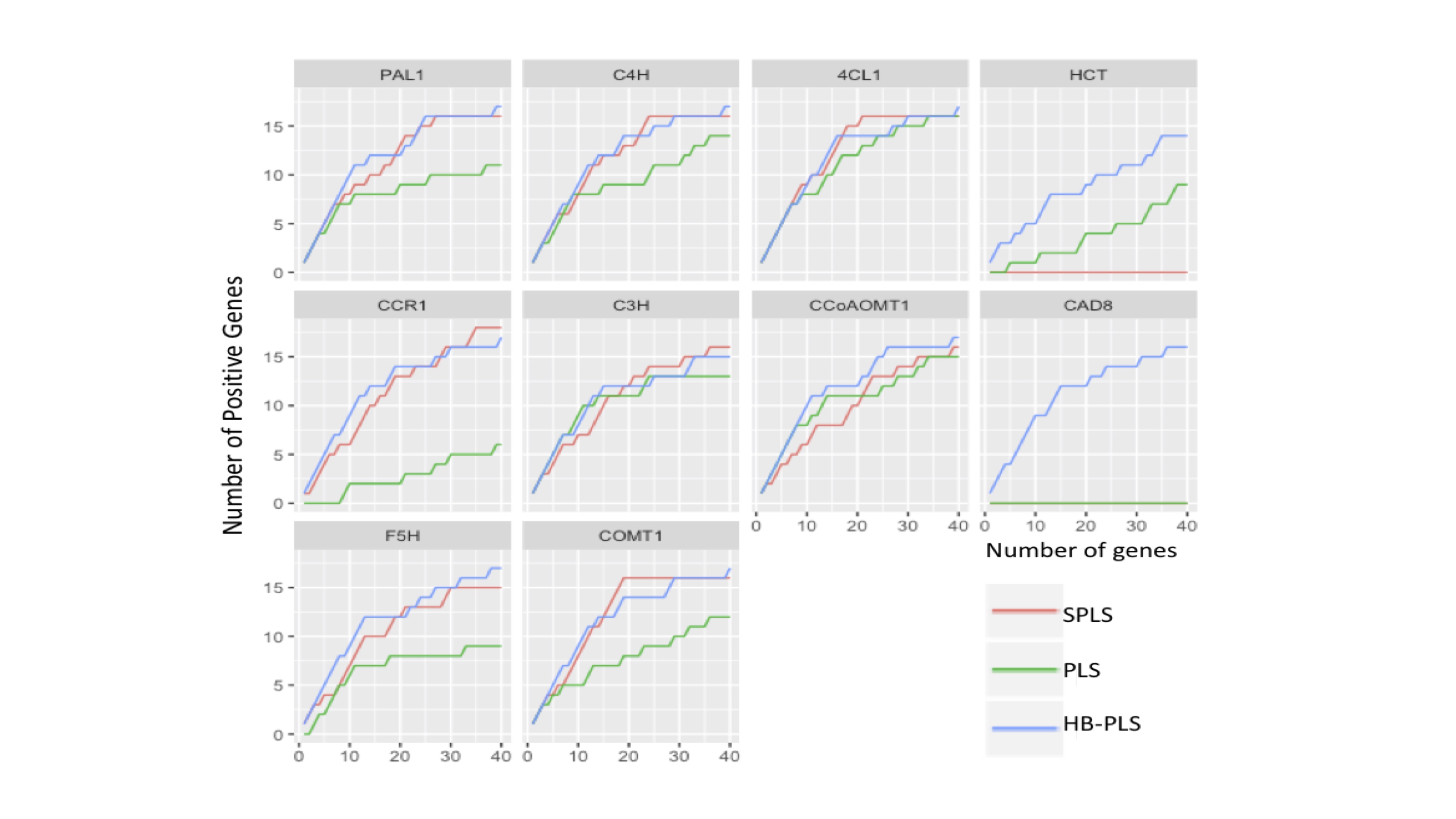

Supplement: Supplementary file 1 — Supplementary data to this article can be found online. [file FR-2021-0006-Suppl-FigureS1.jpg]
